# A structural basis for cellular senescence

**DOI:** 10.18632/aging.100074

**Published:** 2009-07-29

**Authors:** Armando Aranda-Anzaldo

**Affiliations:** Laboratorio de Biología Molecular, Facultad de Medicina, Universidad Autónoma del Estado de México, Paseo Tollocan y Jesús Carranza, Toluca, Edo. Méx., México

**Keywords:** DNA loops, hepatocytes, matrix attachment regions, nuclear matrix, tensegrity, TOR

## Abstract

Replicative senescence (RS) that limits the proliferating potential of normal eukaryotic cells occurs either by a cell-division counting mechanism linked to telomere erosion or prematurely through induction by cell stressors such as oncogene hyper-activation. However, there is evidence that RS also occurs by a stochastic process that is independent of number of cell divisions or cellular stress and yet it leads to a highly-stable, non-reversible post-mitotic state that may be long-lasting and that such a process is widely represented among higher eukaryotes. Here I present and discuss evidence that the interactions between DNA and the nuclear substructure, commonly known as the nuclear matrix, define a higher-order structure within the cell nucleus that following thermodynamic constraints, stochastically evolves towards maximum stability, thus becoming limiting for mitosis to occur. It is suggested that this process is responsible for ultimate replicative senescence and yet it is compatible with long-term cell survival.

## Introduction

Cells of higher eukaryotes cultured *in vitro* complete a limited number of cell divisions and then enter a state of growth arrest that is termed replicative senescence [1,2]. This process has been linked to organism ageing, tumour suppression or terminal differentiation. Indeed, both the post-mitotic state characteristic of fully differentiated cells such as neurons and cardiomyocytes, and the cell-cycle arrest in senescent cells are remarkably stable [3]. This poses the question of how such a long-term stability is achieved. At first glance replicative senescence (RS) seems to be constituted by two separate phenomena: on the one hand there is RS related to exhaustion of a certain proliferating potential of the cell, this has been linked to some sort of counting mechanism that determines the number of completed cell cycles before triggering replicative senescence [4]. On the other hand, there is a stress-induced premature RS that can be triggered by a number of cell stressors such as hyperoxia, DNA damage causing replicative stress, and oncogene hyper-activation, such a RS is now termed STASIS (stress or aberrant signalling-induced senescence) so as to distinguish it from RS linked to the number of cell divisions [5,6].

Telomeres, the capping ends of chromosomes, shorten after each cell division in organisms lacking the enzyme telomerase in adult somatic tissues. Such is the case in humans and non-human primates in which critical telomere shortening correlates with a form of RS [5,6]. However, telomere length is heterogeneous in the human population and shorter lengths do not always correlate with tissue ageing although it appears that telomere-dependent RS may occur in response to the shortest telomere in the cell [5,7]. Cells from other mammalian species such as rodents and lagomorphs (rabbits, hares, pikas) do not show telomere-dependent RS *in vitro*, since the telomeres in these animals are much longer than human telomeres and at least in the case of rodents they express telomerase in adult somatic cells. Indeed, both rodent and lagomorph cells do not display RS in culture provided that culture conditions are optimized [6,8].

STASIS occurs in murine cells in culture and this process is dependent on the expression of the cyclin-dependent kinase inhibitor p16INK4a that keeps the pRB cell cycle regulator in its hypo-phosphorylated state able to repress progression of the cell cycle. Thus such cells are arrested in G1. This arrest is reverted by inactivation of pRB indicating that continued activity of pRB is necessary for maintaining STASIS in murine cells [3]. STASIS in human cells is also triggered by p16INK4a, yet such a process is not reverted by inactivation of pRB or p53 although such an inactivation enables senescent cells to reinitiate DNA synthesis but they cannot complete the cell cycle, suggesting that the cells become arrested in either G2 or M phase of the cell cycle [9,10]. Moreover, human fibroblasts in culture show mixed RS as some cells display telomere dysfunction while others arrest due to spontaneous p16INK4a induction [6]. Actually the INK4a/ARF locus is normally expressed at very low levels in most tissues in young organisms but becomes highly expressed with ageing [11].

Thus there are short and medium lived species (mice live around two years in the lab, while rabbits live some 20 years) that apparently do not display telomere-dependent RS and only display STASIS, while a long-lived species (humans) displays both. However, in the case of humans, the proliferating potential of cells *in vitro* shows a great degree of variability among fibroblasts strains of different humans, even when matched for tissue of origin and donor age, and such a potential can be significantly augmented by manipulating the culture conditions. Also, the proliferating capacity in culture may vary with the cell type [6]. So far the attempts for linking the cellular proliferating potential *in vitro* with both organism's longevity and senescence have produced rather ambiguous results [12]. Indeed, cellular replicative capacity correlates with organism body mass and not with longevity, while telomerase activity seems to co-evolve with body mass and not with lifespan [13,14]. Moreover, fibroblasts from human nonagenarians display a high-replicative capacity in culture [15].

### Is the Hayflick limit an *in vitro* artefact?

The current evidence does not support a relationship between longevity and cellular replicative capacity in culture, yet it suggests that cellular proliferating potential is related to tissue repair and maintenance capacities of the organism, and as such it may have some relevance to the ageing process [6]. However, if we consider that short-lived animals like mice are unlikely to age in the wild, since in wild mice populations 90% mortality occurs by 40 weeks of age, even in the absence of predation [16,17], it then seems rather odd that mouse cells display an apparently unlimited proliferating capacity *in vitro* under appropriate culture conditions in which oxygen is reduced to physiological levels [8,18]. Indeed, even human fibroblasts proliferate much longer when cultured under defined conditions (reviewed in [6]). Moreover, serial transplantation studies indicate that adult mouse hepatocytes have stem-cell-like regenerative potential evidenced by their ability to undergo at least 87 population doublings *in vivo* [19]. Thus we may ask whether the Hayflick limit for the proliferating capacity of normal cells [20] is just a laboratory artefact and in the end Alexis Carrel was right: the cells of a mortal metazoan are intrinsically immortal [21], or whether there is a deeper cellular process, occurring in all kind of metazoans and in most kinds of metazoan cells, that truly and finally limits the replicative capacity of normal individual cells.

For addressing this question let us consider the fact that both RS and STASIS are non-reversible at least in human cells [3,5] and yet RS can by bypassed in human tissues with proliferating potential by a number of mechanisms such as reactivation of telomerase, leading to cell immortalization as a precondition for tumorigenesis [5,22]. It is a fact that malignant tumours can only arise in tissues with proliferating potential hence tissues with a large proportion of post mitotic cells such as the brain and the heart are rarely the seat of malignant tumours and the tumours derived from such tissues arise from cells with proliferating potential like the brain glia or the vascular endothelium [23,24]. Thus cardiomiocytes and neurons are not known to give origin to malignant tumours in adult organisms, and yet both neurons and cardiomyocytes are long-living post-mitotic cells. Moreover, organisms mainly constituted by post-mitotic cells do not develop cancer. For example, tumours in *Drosophila melanogaster* only may arise before the larval stage, thus from cells that preserve a proliferating potential and as such are not terminally differentiated. Adult flies subject to mutagenic ionizing irradiation do not develop cancer [25-27]. This fact indicates that there is no set of somatic gene mutations able to revert the post-mitotic state and so that the post-mitotic state is on the one hand highly stable and on the other hand it cannot be dependent on the continued action of soluble factors acting in *trans* (such as p16INK4a or pRB that trigger or maintain STASIS), otherwise in post-mitotic organisms widespread genome mutagenesis by non-lethal ionizing radiation would be likely to cause inactivation of genes coding for the soluble factors that may act as repressors of cell proliferation, leading at least in some cases to eventual re-entry of formerly post-mitotic cells into the cell cycle.

### Evidence for a third kind of replicative senescence

As mentioned before, there is good evidence that a counting mechanism related to the number of cell doublings and DNA replication is involved in limiting the proliferating potential of cells and that telomeres participate in such a mechanism, but this has only be demonstrated in a limited number of mammalian species such as primates while its absence in other species argues against the universality of the telomere-driven mechanism. However, single-cell cloning studies with normal human fibroblasts revealed a bimodal distribution in the replicative potential of clonally derived cells, indicating that there is a stochastic loss of cell proliferating potential [28-30]. Hence besides the cell-division counting mechanism a process with strong stochastic features is at work in limiting cell proliferating capacity. Moreover, a purely stochastic process, consisting of a sufficiently large number of independent events could mimic the apparently deterministic counting mechanism [2]. Indeed, cultures of normal human fibroblasts are known to be heterogeneous with respect to their ability to divide and to synthesize DNA, and the number of cells unable to synthesize DNA or divide increases exponentially with the age of the culture. So there was a large variation in population doubling potential among the clones isolated from a single mass culture, only about 50% of the clones were capable of more than eight population doublings (PDs) and this percentage was further reduced when clones were isolated from mass cultures at higher PDs. Thus, mass cultures appear to be composed of two subpopulations, one with a low population doubling potential (PDP) and the other with a higher PDP [28]. That a large proportion of cells in a young culture are capable of only a few additional PDs indicates that there is a large variation in the number of divisions which normal fibroblasts can undergo and that the mechanism which establishes the finite in vitro life-span would not be simply the number of cell divisions. The subpopulation of single cells having low PDP increases with increasing PDs of the mass culture at the time of cloning, yet in principle it should be expected that all of the cells with low PDP would be eliminated from the mass culture within 10 PDs, but this is not the case suggesting that cells are recruited into the low PDP subpopulation as the mass culture undergoes more PDs. These facts suggest that a stochastic process is involved in establishing the finite life-span of cells in culture, but this process is not related to telomere erosion as a function of the number of cell divisions.

The adult hepatocytes are cells that rarely divide and it is assumed that they are arrested in G0. However, in young adult rats partial hepatectomy leads to liver regeneration inducing the synchronous entry into the cell cycle of some 97% of the residual hepatocytes, with subsequent return to quiescence of the hepatocytes after liver regeneration. Indeed, functional hepatocytes are not terminally differentiated until very late in life, a fact that correlates with progressive reduction of their proliferating potential [31,32]. Therefore, there is a progressive reduction in the proliferating potential of the hepatocytes as a function of age, and in older animals the percentage of residual hepatocytes able to re-enter the cell cycle after partial hepatectomy is significantly reduced [33]. This fact indicates that loss of cell proliferating potential *in vivo* is not directly linked to a cell-division counting mechanism (and certainly not to telomere erosion since rats have very large telomeres) and that a stochastic mechanism that limits the proliferating potential occurs even in cells that are arrested in G0.

### Nuclear organization and replicative senescence

It has already being suggested that long-term proliferation of normal cells depends upon the potential for reorganization of the genome as a self-limiting process, since at each cell division residual quantitative and qualitative changes would accumulate in chromatin, limiting the long-term potential for further rearrangements [34]. Indeed, during serial replication of normal fibroblasts the cell population undergoes a succession of subtle changes in the initiation of and in the transit through the cell division cycle, rendering the cell population progressively more heterogeneous and finally in the last stage where cells perform their last mitoses there is an abrupt disorganization of cell proliferation followed by a post-mitotic state of indeterminate duration [35,36]. The last mitoses are characterized by a chaotic behaviour in the distribution of DNA between daughter cells, indicating major alteration of mitosis and karyokinesis that involves nuclear disassembly and reassembly. Among the abrupt events seen at this stage is the destabilization of nucleosomes and the decondensation of heterochromatin, as well as the disorganization of the 30nm chromatin fibres [35]. During these chaotic divisions the cell morphology changes dramatically: the cell size increases, the cytoplasm is stretched and less mobile, and the nucleus enlarges. Indeed, almost 100% nuclei enlarge and become abnormally clear while chromatin displays a highly dispersed pattern, indicating widespread heterochromatin de-condensation [35,36]. This is consistent with the heterochromatin loss model of cell ageing (HLMCA) that suggests there is a net loss of heterochromatin with age [37]. The switch to a majority of cells with these ultra-structural characteristics is a sudden phenomenon, as is the rapid decline in the number of cells capable of responding rapidly to growth factors [38]. Thus during the last mitoses fibroblasts go through a final, sudden chaotic state that involves different levels of DNA organization, and this occurs together with an abrupt modification of cell morphology and disorganization of the cell cycle.

### Higher-order structure in the cell nucleus

In the interphase, nuclear DNA of higher eukaryotes is organized in supercoiled loops anchored to a nuclear substructure commonly known as the nuclear matrix (NM) that is a non-soluble complex of ribonucleo-proteins obtained after extracting the nucleus with high salt and treatment with DNase [39,40]. The exact composition of the NM is a matter of debate as some 400 proteins have been associated with this structure [41]. However, apparently there is a limited set of proteins common to the NM from all mammalian cell types [42]. DNA is anchored to the NM by means of non-coding sequences of variable length known as matrix attachment regions or MARs. Yet there is no consensus sequence for *a priori* identification of MARs although they are generally rich in AT and repetitive sequences, and map to regions where the DNA is intrinsically curved or kinked and has a propensity for base unpairing [43]. MARs are classified in structural-constitutive, resistant to high-salt extraction and transient-functional, non resistant to high-salt extraction [43,44]. The higher-order structure of interphase and metaphase chromosomes is likely to be maintained by constitutive MARs [45], and there is evidence that elements of the NM participate in the formation of the chromosome scaffold that constitutes the structural core of mitotic chromosomes [46-48]. In this case the strong interaction between MARs and the insoluble proteins of the NM protects these sequences from high-strength ionic buffers and nuclease digestion [43,44]. However, not all potential MARs are actually bound to the NM constituting true loop attachment regions or LARs [49]. It has been estimated that in a typical mammalian genome the average density of potential MARs is 1 MAR/30 kbp [50]. Thus for example, considering that the haploid rat genome size is some 2.75 Gpb then there should be some 180,000 potential MARs in the diploid rat genome. However, the average DNA-loop size in young-adult rat hepatocytes is 80 - 90 kbp [51] and this figure is compatible with an actual total of some 66,000 DNA loops per rat diploid genome, indicating that the actual number of LARs in the young rat is roughly one third of the potential MARs present in the genome [52]. Therefore, why not all MARs are bound to the NM? There is evidence that when multiple copies of a specific MAR are present these are used in a selective fashion, indicating adaptability of the MAR sequence to serve as anchor only under certain conditions [53]. It has been suggested that dynamic selectivity in the use of MARs as DNA anchors would modulate both the DNA loop average length and the stability of the topological relationships between DNA and the nuclear substructure during development and cell differentiation [54,55].

**Figure 1.**
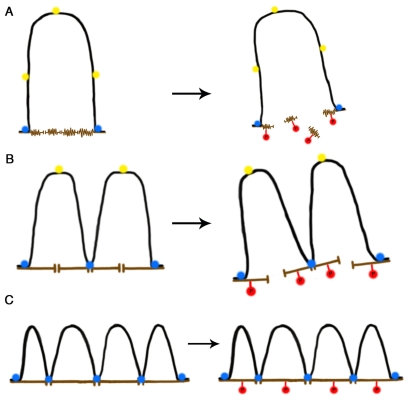
A self-stabilizing tensegrity model for DNA-NM interactions in the cell nucleus as a function of age. (**A**) In a newborn cell NM proteins are in a compacted immature state (brown), thus the NM contact surface is reduced and so a large DNA loop (black) is anchored to two NM segments by means of two MARs that became actual LARs (blue circles) while three potential MARs (yellow circles) cannot attach to the NM due to steric hindrance and lack of enough contact surface. During mitosis biochemical modification of NM proteins (e.g., phosphorylation, red circles) cause disassembly of the NM network leading to disappearance of the cell nucleus. (**B**) In an adult cell the NM proteins are in a more extended state offering a larger contact surface, thus further potential MARs become actualized as LARs reducing the average DNA loop size and increasing the DNA-NM interactions. Yet phosphorylation of NM proteins leads to nuclear disassembly during mitosis. (**C**) In a senescent cell the NM proteins are fully extended thus offering enough contact surface for several potential MARs to become actualized as LARs since steric hindrance is further reduced. DNA loops become shorter on average and DNA-NM interactions are significantly more numerous. Phosphorylation of NM proteins during mitosis cannot lead to nuclear disassembly since the DNA-loops keep separate NM segments bound together and stabilized by means of the LARs attached to the NM. Thus the available energy becomes limiting for disassembling the nucleus and the cell cannot enter or perform mitosis.

Throughout the years only a very limited number of specific proteins have been identified that participate in binding of DNA to the NM in a sequence-specific fashion [40], such proteins are likely to be involved in transient-functional DNA-NM interactions. However, given that there are no MAR consensus sequences and yet the structural DNA-NM interactions occur on a grand scale (for example, saturation experiments indicate the existence of some 150,000 salt-resistant DNA binding sites per NM, [56]), these facts imply that such interactions are the result of indirect readout effects between DNA and NM proteins thus not equivalent to the direct readout interactions between transcription factors and specific DNA-functional groups. Such protein-DNA indirect readouts depend on DNA shape (that is also dependent on nucleotide sequence) and overall DNA mechanical properties [57]. Thus, within the eukaryotic genome there are non-coding sequences with a broad range of affinities for potential attachment sites at the NM, as well as in the NM there are structural proteins with a broad range of affinities for potential MARs. A model for explaining how such mutual affinities are regulated and actualized suggests that the binding of MARs to the NM will depend on three basic factors: first, the degree of mutual affinity between the DNA sequence and the potential NM attachment site. Second, the degree of steric hindrance imposed by the relative density of potential attachment sites per unit length of NM and the limited deformability (stiffness) of the DNA resulting from its persistence length [55]. Such a persistence length is actually dependent on nucleotide composition [58]. Third, the degree of structural stress along the DNA fibre that modulates the overall deformability of DNA that is a compromise among bending, opening, uncoiling or breaking up. Hence the very same DNA sequence can be stably attached to the NM or not depending on the three above-mentioned factors.

 Currently there is ample evidence that the cell is a high-wired system able to transduce mechanical information. Indeed, cells within solid tissues are part of a continuum system of mechano-transduction that couples the extracellular matrix, with the cytoskeleton and the cell nucleus [59]. Thus the cell can be modelled as a vector field in which the mechanically linked cytoskeleton-nucleoskeleton may act as coordinated transducers of mechanical information [55]. The concept of tensegrity defines structures composed by continuous tension elements and discontinuous compression elements, in such systems the role of the compression elements is minimized and the force is distributed among tension elements that can be slender and lightweight [60]. There is plenty of experimental evidence that both cell and tissue tensegrity are a biological fact [61,62]. Accordingly, some models predict that permanent changes in cell shape must lead to modified mechanical interactions within the cell and this would lead to structural changes within the cell nucleus resulting in redefinition of DNA loop domains [55]. This has been demonstrated *in vitro* by inducing a stable modification in cell shape that resulted in the establishment of new high-salt resistant DNA-NM interactions and the elimination of some of such previous DNA-NM interactions, suggesting that both cellular and nuclear shape may act as cues in the choice of potential MARs that should be actualized as LARs [63].

### Evidence for a structural basis for replicative senescence

The naked DNA loops plus the NM constitute a "nucleoid" and since the loops remain attached to the NM they are topologically constrained and supercoiled even after complete extraction of histones and other chromatin proteins [64,65]. The loop DNA supercoiling is higher in the regions closer to the NM, save for the actual LARs that apparently work as buffers against extreme supercoiling [66]. Supercoiling is a structural barrier against the action of endo-nucleases that hydrolyse the DNA backbone by a single-strand cleavage mechanism such as DNase I [67]. Also, in such nucleoids the regions of DNA located close to the NM are relatively protected from endonuclease action by being immersed in the matrix framework that may act as a physical obstacle [44,68]. Digestion experiments with DNase I using nucleoids from freshly isolated rat hepatocytes indicated a progressive slow-down in the kinetics of nucleoid-DNA digestion as a function of animal age. This suggests that a larger fraction of nuclear DNA gets closer to the NM with time [52]. On the other hand, titration with increasing concentrations of the DNA-intercalating agent ethidium bromide (EB) monitors both the integrity and supercoiling of the DNA loops [64,69]. The EB acts as a molecular lever causing the unwinding of loop DNA that produces a halo that surrounds the NM, this process induces tearing forces as the DNA rotates and expands during unwinding, such forces impinge upon the NM as DNA is anchored to it. In nucleoids from newborn (P0) and baby (P7) rat hepatocytes the forces liberated by the EB-induced DNA unwinding lead in the first case to complete disintegration and in the second to severe fracturing of the NM framework. However, in nucleoids from young adult (P80) and senescent (P540) rat hepatocytes the EB releases the DNA loops creating well-defined DNA halos that surround the undisturbed NM framework, yet the average halo size is significantly reduced with age. The average DNA halo size has been correlated with the average DNA-loop size [68,70], and so it was possible to estimate that the hepatocytes from senescent rats have on average a smaller DNA-loop size than in young adult rats: 31 and 48.9 kbp from tip to base, respectively [52].

The protein composition of the rat hepatocyte NM shows no significant qualitative difference as a function of animal age. However, the NM undergoes quantitative changes of the major constituent proteins such as lamins A, B and C, as well as changes in the ratios of such proteins [52,71], this is consistent with previous studies comparing the NM of young and aged human fibroblasts, using 2D electrophoresis [72]. The noticeable increase with age of the three nuclear lamins seems to be relevant to the obvious strengthening of the NM with age [52]. Indeed, micromanipulation methods show that nuclei in human embryonic stem cells are highly deformable and stiffen 6-fold through terminal differentiation, while nuclei from adult stem cells possess and intermediate stiffness. Knocking down lamin A/C in differentiated epithelial cells leads to nuclear deformability similar to that of the adult stem cells [73].

There is an average increase in diameter and volume of both the nucleus and the NM in hepatocytes from senescent rats (P540) and so the NM framework becomes proportionally larger with age [52], this correlates with the reported increase in nuclear roundness with age that smoothes out the invaginations of the nuclear contour [72]. The mean compactness of the NM proteins decreases during development being in the adult rat one fourth of that in the 16-day foetus, suggesting that the NM protein network becomes more extended as development progresses [71]. Such a reduction of NM-protein compactness suggest the progressive shift from a nuclear substructure consisting of unconnected, merely clustered ribonucleoprotein (RNP) fibres and granules to a mature, continuous internal network consisting of interconnected and branched RNP filaments that connect to the nuclear lamina [39,74,75]. A reduction in NM protein compactness or smoothing-out of NM curvature, together with an increased NM volume would also make available a larger contact surface for potential MARs that may bind to the NM depending on their affinity for NM proteins and the degree of local steric hindrance resulting from compactness or extension of the NM protein framework. The HLMCA model proposes that eudomains are the default state for chromatin and that the epigenetic heterodomains are metastable and thus prone to decay into less folded chromatin structures [37] and so there is evidence that heterochromatin is much reduced in the nuclei of aged cells [35,36]. Thus, in nuclei from aged cells virtual MARs formerly occult within heterodomains may become available for interacting with the NM establishing further DNA loops.

The following observations: reduction of loop DNA sensitivity to DNase I, reduction of the average DNA-loop size, increase in the nuclear volume and reduction of nuclear deformability with age [52,73], correlate with the known reduction of the cell proliferating potential with age, even when cells have not undergone repeated cell division cycles through the years (as in the case of quiescent rat hepatocytes, [33]). Thus, the experiments with rat hepatocyte nucleoids indicate that in nuclei from aged animals there is a larger number of DNA-NM anchoring interactions, resulting in a larger number of DNA loops that are significantly shorter and more stable than those in nuclei from younger-animal cells, and so the actualization of potential DNA-NM interactions increases with time.

An important question is what could be the driving factor behind the post-natal increase in nuclear size and volume that establishes the basic condition for further consolidation of the DNA-NM interactions. A typical feature of senescent cells is that they are large-sized (hypertrophic), also it is well known that cell size increases in culture as cells progress toward senescence [76]. Moreover, the liver is an organ that keeps growing during the post-natal period but all evidence suggests that this growth is primarily by hepatic hypertrophy that correlates with a trend of the hepatocytes to undergo polyploidization as a feature of cell maturation. In the liver of normal young rats already 60% of hepatocytes are mononucleated polyploid cells [77], indicating that DNA synthesis has been proceeding in absence of both karyokinesis and cytokinesis; and in older rats there is a direct correlation between higher prevalence of polyploid cells and increasing age [78]. There is evidence that the onset of polyploidy in hepatocytes is associated with weaning and assumption of independent feeding in rodents and that the insulin/Akt pathway is involved in the control of this process [78,79]. Interestingly, the nutrient-sensing TOR pathway that is activated by insulin, growth factors and nutrients is an essential controller of cell growth. TOR (target of rapamycin) is a serine/threonine kinase that participates in two distinct multiprotein complexes (TORC1 and TORC2) each of which signals through a different set of effector pathways. TOR is conserved from yeast to human and strikingly the inhibition of the TOR pathway prolongs lifespan in yeast, worms, flies and mice [80,81]. Thus it was predicted that blocking the cell cycle without a corresponding block of cell growth would cause cell senescence and this has been experimentally confirmed *in vitro* since when the TOR pathway was active and the cell cycle was blocked cellular senescence occurred [82]. This important result ties in with recent evidence *in vivo* that the insulin/Akt pathway directly or indirectly through TORC2, is involved in the process that leads to generation of polyploid hepatocytes in rodents [79], suggesting that growth and aging may share a common molecular mechanism [83].

From the structural perspective, the topological organization of higher-order DNA structure based on selective use of a limited set of potential MARs (as seen in nuclei from newborn and baby animals) is highly asymmetrical and the natural trend for most physical systems is towards reducing the asymmetries in such a way that the system evolves in time so as to become more symmetrical [84-86]. A topological configuration in which most potential MARs are actually bound to the NM, thus resulting in shorter and more stable DNA loops, is also a more symmetrical structural attractor. Moreover, since entropy is not a measure of disorder or chaos, but of energy diffusion, dissipation or dispersion in a final state compared to an initial state [87], such a highly-stable DNA-loop configuration satisfies the second law of thermodynamics since the structural stress along the DNA molecule is more evenly dispersed within the nuclear volume by increasing the number of DNA-NM interactions (thus increasing, in terms of molecular thermodynamics, the occupancy of more microstates in phase space). A larger number of DNA-NM interactions create a structural complex, similar to a hanging bridge in which beams (proteins) and tensors (DNA) interact for creating a highly stable overall structure. Thus any relatively stable interaction between two NM-protein filaments will be further stabilized if a given DNA loop interacts with both filaments, but also the stability of the DNA loop shall be increased by the interaction with both protein filaments, resulting in a self-reinforcing structural stability that operates at the scale of the whole interphase nucleus (Figure 1).

However, there are some terminally differentiated cells whose post-mitotic stage is rather short-lived (in the order of days). Indeed, for such cells terminal differentiation is the antechamber of cell death. Such is the case of lymphocytes, neutrophils, sperm cells or epidermal cells, all of which have very limited life spans after terminal differentiation and either do not constitute solid tissues or are located close or at the open edge of a solid tissue. In such cells there is limited scope for tissue mechano-transduction acting as guide for nuclear organization. Interestingly, in these cells terminal differentiation is linked to induction of DNA strands breaks that preferentially occur at sites involving MARs, liberating DNA fragments of some 50 kbp, that roughly correspond to the average distribution of chromatin looped domains [88]. Indeed, ribo-nucleoprotein-masked nicks exist in the genome distributed on average every 50 kbp, suggesting that eukaryotic genomic DNA is composed of contiguous rather than continuous single strands, interrupted at the boundaries of interphase chromatin loops [89]. This fact supports the notion that attachment to the NM contributes to stabilize the long-range DNA structure. On the other hand, massive breaking of DNA in regions corresponding to actual LARs would cause inability to perform appropriate chromosome condensation during mitosis as well as to complete nuclear reassembly. Several important processes of nuclear physiology, such as replication, transcription and processing of primary transcripts occur at macromolecular complexes located upon the NM [90-92]. Thus the topological relationship between DNA loops and the NM is very important for appropriate nuclear physiology. For example, productive infection by herpes simplex virus type 1 induces DNA breaks and wholesale alteration of higher-order structure of the host cell chromatin, resulting in loss of DNA-loop supercoiling and organization that correlates with complete inhibition of host-cell replication and transcription [69,93-95]. Indeed, correct repair of DNA damage must include the recovery of both the double helix integrity and the complex third-dimensional DNA topology, otherwise the cell will not survive [96,97]. Therefore, cells with overall disruption of higher-order DNA structure are irreversibly committed to functional failure in the short term.

### Why a stable higher-order nuclear organization leads stochastically to replicative senescence

Highly stable physical systems are quite resistant to change and have a much reduced dynamic potential. Thus, a structurally-stable cell nucleus would not be the seat of both the dynamic transitions necessary for mitosis and the rearrangements of chromosome territories and chromatin domains in early G1 [98] that normally occur in cells with a positive proliferating potential, since the energy cost of nuclear disassembly and reassembly will be limiting for the cell. Indeed, the sub-nuclear organization of interphase chromosomes in pre-senescent mammalian cells is quite different from that in proliferating or quiescent cells, indicating that on average the spatial organization of the genome within the nucleus changes with age [99]. Thus the nuclear higher-order structure established by the topological interactions between chromatin and NM constitutes an integral structural system that naturally but relentlessly evolves towards a more symmetrical and highly stable state. Since this process obeys thermodynamic constraints it must follow a stochastic behaviour that nevertheless increases its probability as a function of time. This might be a more general, physical basis for terminal, non-reversible cell differentiation, leading to cellular replicative senescence and a long-lasting, highly stable post-mitotic state that is independent of the action of soluble factors acting in *trans* and that occurs in a stochastic but time-dependent fashion within cell populations, whether or not the affected cell has previously divided (thus independently of any cell-division counting mechanism).

Heterochrony is developmental change in the timing of events, leading to changes in size and shape. There is no doubt that during embryogenesis there are changes in the rate or timing of development of some cell lineages in the body relative to others, so that different cell lineages develop at different rates. Mechano-transduction during tissue morphogenesis may induce changes in the differentiation state of cells and such a modification of the differentiation state also impinges on the potential morphogenetic trajectory by limiting the repertory of changes in cellular size and shape. Heterochrony may alter the distribution of probabilities of stochastic events such as the rate of actualization of DNA-NM interactions, hence some cell types such as neurons reach terminal differentiation and became post-mitotic earlier than others, depending on their morphogenetic trajectory. As a corollary it can be concluded that such a highly-stable nuclear post-mitotic structure cannot be altered, reverted or bypassed by any known oncogenic stimuli and as such is the true barrier against tumorigenesis.
